# S4 prescribing for podiatrists

**DOI:** 10.1186/1757-1146-4-S1-I5

**Published:** 2011-05-20

**Authors:** Stephen Marty

**Affiliations:** 1 Pharmacy Board of Australia, PO Box 9958, Melbourne, VIC, 3001, Australia

## 

This session will cover the legislation affecting the prescribing of scheduled medicines by endorsed podiatrists, possession, administration and supply. It will include the differences between state and territory drugs and poisons requirements, issues in the preparation of prescriptions and what can go wrong. Risk management and the role of podiatrists in the quality use of medicines in Australia will also be discussed.

